# Integration of primary health services: being put together does not mean they will work together

**DOI:** 10.1186/1756-0500-7-66

**Published:** 2014-01-30

**Authors:** Sharon Lawn, Andrea Lloyd, Alison King, Linda Sweet, Lyn Gum

**Affiliations:** 1 Flinders Human Behaviour and Health Research Unit, Flinders University, Margaret Tobin Centre, Adelaide, South Australia, Australia; 2 Flinders Innovations in Clinical Education, Health Professional Education, Flinders University, Adelaide, South Australia, Australia; 3 GP Plus Marion, SA Health, Oaklands Park, South Australia, Australia; 4 Flinders Innovations in Clinical Education, Flinders University Rural Clinical School (FURCS), Flinders University, Adelaide, South Australia, Australia; 5 Flinders Innovations in Clinical Education, Flinders University Rural Clinical School (FURCS), Flinders University, Renmark, South Australia, Australia

**Keywords:** Interprofessional practice, Territorialism, Co-location, Community mental health, Primary health care

## Abstract

**Background:**

This paper reports on an Australian experience of co-locating a range of different primary health services into one building, with the aim of providing integrated services. It discusses some of the early challenges involved with moving services together and reasons why collaborative and integrated working relationships to improve the clients’ journey, may remain elusive.

**Methods:**

Ethnographic observational data was collected within a GP plus site as part of day-to-day interactions between the research officer and health professionals. This involved observations of team processes within and across teams at the site. Observations were thematically analysed using a social anthropological approach.

**Results:**

Three main themes arose from the analysis: Infrastructural impediments to collaboration; Territorialism; and Interprofessional practice (IPP) simply not on the agenda. The experience of this setting demonstrates that dedicated staff and resources are needed to keep IPP on the agenda of health service organisations. This is especially important where organisations are attempting to implement new models of collaborative and co-located services. Furthermore, it shows that establishing IPP within newly co-located services is a process that needs time to develop, as part of teams building trust with each other in new circumstances, in order to eventually build a new cultural identity for the co-located services.

**Conclusions:**

Co-located health service systems can be complex, with competing priorities and differing strategic plans and performance indicators to meet. This, coupled with the tendency for policy makers to move on to their next issue of focus, and to shift resources in the process, means that adequate time and resources for IPP are often overlooked. Shared interprofessional student placements may be one way forward.

## Background

This paper reports on an Australian experience of co-locating a range of different primary health services into one building, with the aim of providing integrated services. It discusses some of the early challenges involved with moving services together and reasons why collaborative and integrated working relationships with each other, to improve the clients’ journey, may remain elusive, despite being one of the intentions of the move to a single site.

### Description of the setting

The setting is a GP Plus Health Care Centre; a community health service with over 250 community healthcare staff from a range of resident agencies including primary health care, mental health (adult and youth), dentistry, allied health, pathology, and youth services; and visiting services including sexual health, drug and alcohol counselling, chronic disease and medical outpatient clinics. The building of GP Plus Centres is part of the South Australian Department of Health’s [1] reform agenda to strengthen primary health care services. The main goal of this initiative is to present a complete system of health care to clients, integrating existing primary health care services and community mental health services with General Practice and non-government services. Together, these services aim to provide coordinated care, including to people with chronic and complex conditions, and people with co-existing mental and physical illness [1]. This approach to service delivery is in keeping with similar strategies in other countries [2,3]. Furthermore, these Centres are intended to support “increased teaching, training and education opportunities for health professionals” [1] (p.6), by providing infrastructure for clinical placement opportunities, and the reorganisation of the workforce through, “the development of multi-skilled teams through standardised training and skill-based competencies” [1] (p.11).

The GP Plus Centre is a purpose-built suburban site, located next to a major retail centre, and service and transport hub. A Service Development Group with key stakeholder representatives was formed in 2006 to inform design and development of the Centre prior to construction commencing. An intensive consultation process was undertaken, involving the agencies identified as willing to move into the Centre, their staff and clients, experts in local population needs, plus other service providers and community members in the region. The Centre was completed and opened in May 2011. In this period, there were some changes to the configuration of resident agencies to be accommodated within the building. The most significant of these was the inclusion of a new Community Mental Health Centre, with final approval being given for this after construction of the GP Plus Centre had commenced.

The Centre is designed around client groups and clinical areas, rather than individual agencies, as per the agreed design principles [1]. Client service delivery is concentrated on the two lower levels of the building, with non-client activity on the two upper levels of the building. The ground floor accommodates facilities and consultation areas for children and families, youth, mental health and the main Centre reception. The Centre has one main entrance, a design feature intended to support integrated service delivery, with a secondary and less conspicuous entry to the youth area, in response to community consultation. Level One (ground floor) accommodates a single large reception, the dental service, two consultation areas mainly for adult services with 24 consultation rooms, including specialty rooms for podiatry and audiology, and a separate mental health reception that was established later. Consultation zones are made up of six clinical and counselling rooms, supported with a team room. Group rooms and a rooftop garden for group health programs are on the second floor, along with non-consulting workspaces accommodating primary health, youth, and child and adolescent mental health services. The third floor provides a similar arrangement of non-consulting workspaces and office space for adult mental health services. The non-consulting workspaces consist predominantly of large open workstation spaces and some offices. The separation of consulting space from workstation space for non-client related activities was a requirement of the state government office accommodation guidelines, and was a completely new way of working for some of the teams. To assist in the transition to this new way of working, the allocation of workstations was based on a ‘hoteling’ model, with teams being located together in the same area. Hoteling refers to the use of a set of desks or cubicles for workers who come in to the office from time to time, where the desks are booked ahead of time. It involves unassigned seating in an office environment, with provision of quiet spaces intended for conversation or extended phone discussions [4,5]. An adaption of this hoteling model meant teams were assigned areas of desks and were able to book staff in to a regular desk space for the sessions they needed. On each floor, senior staff have individual offices located around the periphery to promote availability to their team. Elevators and an open stairwell provide the access between floors and also to the car park, offering staff direct access to each floor of the building. Clients have direct access to the relevant areas for client consultation via the main entrance or youth entrance.

### The project

The research project, during which the challenges reported here were observed, was established and jointly funded as an interprofessional education (IPE) and interprofessional practice (IPP) project between Flinders University Faculty of Health Science academics, Southern Adelaide Health Service (or SA Health) represented by two GP Plus Centre managers, and Southern Knowledge Transfer Program (SKTP), a Flinders University organisation established to build links and partnerships between the university and community agencies. The IPE/IPP project sought to establish a mutually agreed framework of IPP capabilities to improve IPP in the workplace and improve the student placement and transition to employment experiences. The GP Plus community members saw value in developing a capability framework that would inform their planning and development of a collaborative model of care for the new Centre, as well as being a catalyst for change to meet some of the objectives and rationale for the Centres’ development.

## Methods

Ethics approval for the broader study was granted by the Flinders University Social and Behavioural Ethics Committee. This included approval to undertake focus groups, day-to-day interactions with staff and observations within the Centre as part of the ethnographic method. A project steering and research group met monthly throughout the project. In order to straddle the bridge of academia and practice, a research officer was sought with experiences in both community health and academia. She was employed by the University, spending half of her time located at the University and the other half in the Centre, with a desk in the general area of the primary health care team.

Qualitative methodology was used within the overarching theoretical stance of ethnography because the project involved a case study of the particular GP Plus environment. This methodology is useful when studying complex social and behavioural phenomena in detail and from an experiential standpoint for a specific population or setting [6]. Appreciative enquiry informed the theoretical questioning that underpinned the research. This approach shifts from attempting to identify ‘What works?’ or ‘Does this work?’ to developing an understanding in depth of ‘What works where, for whom, and in what circumstances?’ It thus takes into account the local conditions in which changes are introduced in the field [7]. The research was ethnographic in that, “its goal [was] to produce a description which allows others to understand the culture from inside in terms that the participants themselves used to describe what is going on” [6] (p.143). Also akin to ethnographic research, the research officer habituated into the site over an extended period of time. This is an important process that improves rigor by fostering circumstances where participants act as they would normally in their setting, not staging behaviour because they know that they are being researched [8]. Habituation to and ongoing presence at the site occurred between June 2011 and March 2012, during which time the research officer engaged in conversations with health workers informally at the site, formally met with individual health workers and managers, developed lunch time educational sessions, and attended resident agency meetings held at the site.

Action based research processes were central to exploring and understanding the dialogues and interactions within the setting [6]. The underlying purpose of the IPE/IPP project, evident in the three focus group cycles (blinded [9] was to inform the model of care for the setting and to develop a shared IPP capabilities framework across the University and the field. This fits with the intention of action research to influence or change some aspect of the focus of the research. As Robson [6] stated, “Collaboration between researchers and those who are the focus of the research, and their participation in the process, are typically seen as central to action research” (p.188).

Further data was collected from focus groups with health professionals (HPs) (clinicians with direct client contact), from across the resident agencies at the site between August and November 2011 (cycle 1 involving 14 HPs, cycle 2 involving 20 HPs). They represented a range of health disciplines including nursing, dietetics, speech therapy, podiatry, lifestyle advice, dental hygiene, youth work, occupational therapy and social work. Similar focus groups were also held with academics across various disciplines at the university. The purpose of the focus groups was to ask what capabilities are needed to work interprofessionally and what barriers exist. Cycle 3 involved two workshop options with HPs from the Centre and academics from the university coming together to verify the capabilities. These groups formed part of the process for developing an IPP capabilities framework, the results of which are reported elsewhere [blinded, [9]. An underlying driver for the project was the improvement of interprofessional experiences for students across the disciplines whilst on placement in the Centre, and university IPE that prepared them better for placement.

Participation in the focus groups was voluntary, consent was sought, and recruitment occurred through an expression of interest email and via posters in tea rooms, as well as incidental ‘would you like to come along’ interactions during day-to-day contact with health workers in the building. There were three initial focus groups with HPs working at the GP Plus Centre during cycle 1; groups one and two were multi-agency and multi-disciplinary, and the third was with the multi-disciplinary chronic disease primary care team that attended at the suggestion of the manager of these services. All data was de-identified to ensure participants’ anonymity.

### Data collection

The research officer collected data as part of day-to-day interactions described above. They observed team processes within and across teams at the site, collating reflective notes in a journal. A predetermined observation schedule was not used. Rather, the research officer made comprehensive reflective notes at the end of each day at the Centre. This included their reflections on the process of establishing the focus groups and general observations of the daily work at the Centre, including observed interactions between agencies and HPs that appeared to impede the progress of the project and the collaborative opportunities it offered. These observations were reported to the combined steering/research group during its monthly meetings. It became apparent to the project team that there were unanticipated barriers to achieving the project’s objectives that were worthy of further research focus. The research project’s progress was impeded by difficulties in engaging staff in the project. This appeared to be a consequence of other behaviours that were emerging within the setting, attributable to the transition of multiple agencies co-locating into the one site. Hence, the data reported here were subsidiary to the original research and discussion of IPP capabilities.

### Data analysis

In order to examine the barriers to achievement of the project and the Centre’s core objectives, the data from the research officer’s journal and focus groups was examined to understand what impediments were observed and reported to achieving these objectives. . Data analysis occurred within a social anthropological approach [10]. This approach was used because we were interested in the behavioural regularities apparent in everyday interactions of health service staff within the setting, comparing and contrasting what they said with observations of what they did. As Miles and Huberman [10] stated, “These regularities often are expressed as ‘patterns’ or ‘language’ or ‘rules,’ and they are meant to provide the inferential keys to the culture or society under study” (p.8). The data was collected, reviewed and analysed manually by the research officer and another member of the research team (who was not an observer, but was experienced with ethnographic methods) for evident themes [8,11]. Together, they undertook extensive discussion and debate about the meaning of the data, within a social anthropological framework. This looked at the observed behaviours of participants within the environment in which they interacted with each other, the physical environment in which those behaviours occurred, and any observed impacts of that environment on those behaviours. These interpretations were supported and contrasted with what focus group participants said about what IPP should look like. Once these researchers agreed on the interpreted themes, further discussion and verification of the themes was undertaken. To do this, the themes were presented to the project’s combined steering/research group at its monthly meetings, where they were discussed, debated and refined.

## Results

From this analysis, two main themes were identified. These were: *Structural Impediments to Collaboration (Subthemes: Managerial/Governance, Physical Infrastructure and Information Management Systems) and Territorialism.* The consequence of these is described in a further and theme as: *IPP Simply not on the Agenda*.

### Infrastructural impediments to collaboration

The purpose of bringing the services together into one location was to foster improved collaboration and working relationships between services [1]. This was considered to be particularly important for caring for clients with comorbid health conditions and those with complex needs [12]. However, a number of features of the infrastructure within the site - governance, physical and technical - were noted to pose barriers to achievement of these goals.

#### Managerial/Governance

There were no changes in the governance of the agencies moving into the Centre once there; that is, each agency/team maintained its own separate lines of governance. Although shared governance is not deemed essential for effective collaboration [13], limited focus on governance meant that the Centre Manager had no authority in how the co-located services operated, communicated and structured their work with each other. This meant that each service moved into the building and brought its existing operational processes and culture with them. To assist with any issues arising from independent governance arrangements, a Resident Agency Managers group was formed, consisting of the manager of each resident service. Variations in information filtering back to the teams from these meetings were observed. This variation likely arose because communication translation relied on the capacity and actions of each of those individuals to communicate the information among their own agencies.

The Centre Manager was in fact manager of the operations of the building only, and fiscal resources were limited to cover the essential building operational costs only. There were no resources available for integration and change management for resident teams. Administrative resources for the Centre were attached to resident teams; therefore, Centre administration was dependent on negotiation and cooperation of the teams. As there was no change in governance structure to aid integration and sharing of operational responsibility, most of what was achieved was done through negotiation and willingness on the part of stakeholders to collaborate. There was no upfront establishment of clear memoranda of understanding between services. Despite agencies possessing a shared vision for the Centre [1], it appeared that they did not possess shared values or understanding of what needed to be done. In the absence of a shared model to guide them, agencies maintained their boundaries. An example is the mental health service erecting a separate sign on the building soon after moving in.

Another example of lack of shared governance, which consequently impeded the achievement of integration goals, related to the use of the crèche facilities. Crèche (child-minding) facilities were constructed to support client access and early childhood development programs in the Centre. The Primary Health service had been providing a funded crèche service in their previous venue. During Centre development there was a shared vision that the crèche would be available for all clients accessing the Centre, regardless of which service they were attending. However, without a governing authority and no other agencies willing to contribute financially, access to the crèche service was limited to clients of the Primary Health Service. Goodwill and negotiation on a case-by-case basis has enabled access for some clients of other services; however, this is less than ideal.

#### Physical infrastructure

The size and structure of the building (7500 square metres over four floors) meant that many services were physically separated from other services by being on different floors. The building was designed in functional zones and, for security purposes, clinical staff and consultation areas were not accessible to the general public. Access to locked areas required the use of proximity cards which were also the staff’s identification cards. Staff in some resident teams had limited access to areas within the building. The requirement to use a proximity card to access functional areas of the building appeared to impede communication between workers across the different services. This was most apparent between mental health services and the other services, as the mental health area had higher security specifications. Consequently, open collaboration was made more difficult because of the layers of locked doors, which discouraged or blocked HPs from making physical contact with HPs in other services in the building.

The structural nature of this multistorey building and its functional zones also meant staff could go in and out of the building via the elevator and stairwells without having any visible contact with staff from other services. Limited chance encounters included sharing the elevator, running into each other in the car park, or seeing each other in other areas of the building. These physical features of the building impacted negatively on staff’s ability to interact spontaneously.

It was believed that the open space design of the office work areas would assist communication within and between teams by providing close proximity without walls as barriers between workers. However, it appeared to have the opposite effect and impeded communication. Workers reported that they did not feel comfortable to partake in social chat in these spaces because they may be perceived as not working, and they did not want to disrupt others’ work. As was mentioned in one focus group, “A large open space can inhibit interaction as opposed to sharing office with one or two”. Another participant stated, “there may be 8–9 people in an open room but some don’t talk to us…there are barriers in an open room”. These HPs alluded to the open plan work spaces as a barrier to the development of effective professional relationships across the Centre. Some studies confirm this concern; whereas, much of the established literature suggests that open plan work environments foster greater collaboration and teamwork [14-16]. The various youth focused services within the Centre did not appear to have such barriers, likely because they were smaller teams and in closer proximity to each other, with shared corridors.

### Information management systems

Shared client information management system software was developed and attained specifically for use in the Centre to enable shared client information across services. It was intended that, over time, the information management systems in the Centre would enable the creation of common client identifiers and records. This was so that, with client consent, all service providers involved in the client’s care would have access to the necessary health information, and also so that clients would only have to provide their details once [1].

Whilst the intent was to promote collaborative practice, the information management systems became another infrastructural impediment to this end. As each resident agency co-located, some teams were required to use different information management systems from each other to maintain their service networks with other sites. Some of the agencies provided a state-wide service and/or had legislative requirements to use a particular system. They were therefore unable to relinquish those systems to use the shared system. This resulted in client information in these systems not being available to workers from other services.

There was no systematic method to ‘flag’ if a client was being seen by other services in the building. This had the potential for clients to have multiple care plans and fragmented care, which was far from the co-ordinated care mission [1]. In addition, there was concern expressed amongst the HPs about what they perceived as unnecessary sharing (or access) of client information which they felt was in conflict with their desire to protect their clients’ privacy. As one focus group participant said, “Is it really necessary for the dental service to be able to see that my client was raped at the age of 14?” Despite the new shared electronic system having greater security than other record systems, and other protection features which did not give all clinicians access to all client notes, there remained considerable work to be done to achieve acceptance of the notion of shared records.

In summary, each infrastructural issue – governance, physical and technological – served to perpetuate the shared challenges created by each issue individually.

### Territorialism

Within days of moving into the Centre, the research officer observed territorial behaviours within the space utilised by each health service, and this continued to build over time. This territorial behaviour was identified as a major barrier to the establishment of the Centre objectives of service providers working together to enable improved coordination and delivery of care [1]. Territoriality is the term used to describe the state which is characterized by possessiveness, control, and authority over an area of physical space. For the need of territoriality to be fully met, the person must be in control of some space, able to establish rules for the space, and to defend it against invasion or misuse by others. The right to do those things must be acknowledged by other persons [17].

During the period of data collection, behaviours were observed including individuals and teams staking personal ownership of spaces within areas by means of signs and labels. This territorial behaviour extended into spaces that were meant to be for shared communal use to promote collaboration. This was particularly evident in the main lunchroom space where one team would ‘reserve’ tables between certain hours of the day, taking ownership of the space, and inhibiting others from using the space at the same time.

In an effort to reduce the territorial behaviours, guidelines were introduced for the use of shared clinical spaces, as well as for the allocation of workstation spaces as described earlier. This was done to standardise rooms for multiple users and provide consistency in bookings for the clients and workers. However, the desire for individuals to create a personal space soon became apparent with workers taking ownership of space, pinning up personal pictures, and installing pot plants and mementos in areas that were meant to be shared.

Interestingly, HPs were observed to overcome their territorialism when they were attempting to serve the needs of complex care clients who were receiving or needed multiple services. Often, provision of care to these clients was highly challenging for each of those services. Under these circumstances, strong collaborative relationships were formed between HPs from different services in order to address the significant shared problems with providing care that confronted each service. Such cases highlighted their shared struggles and frustrations and willingness to communicate regardless of service structures that might impede this. It ensured that they demonstrated highly person-centred approaches, literally to engage such clients more effectively.

### IPP simply not on the agenda

Collaboration and acceptance of the value of IPP had occurred at management levels of each service [1], and continued to be promoted at this level. There were some excellent examples of IPP within teams and within each agency. However, this had not translated to IPP collaboration between agencies, for the benefit of providing holistic and coordinated care to shared clients, except when client complexity was significant, as already noted. Collaboration and IPP intent were clearly evident in strategic reports developed as part of the GP Plus initiative [1]. However, with no strategy or resources to build IPP, overall lack of investment in supporting the change process in a systematic way, and arguably being ill-prepared for the structural and behavioural issues identified in the first two themes, inter-disciplinary teamwork existed within teams but was often not extending beyond them. The capabilities and values required for IPP struggled to move from rhetoric to reality in the face of these more structural processes, undermining the development of a more collaborative IPP model of care. Another important issue was the sense of imposed change for some services in the building; that is, some services had moved into the Centre reluctantly under direction from the state health service and did not desire or intend to change their practices.

In the planning phase for the Centre, much attention was given to how services would work together. However, for the first few months following the move into the Centre, the Resident Agency Managers were consumed with the practical aspects of moving into a new building, rather than developing interagency collaborations and values through a model of care that would underpin this. Practical issues were prioritised. These included fire and emergency procedures, personal safety and security, occupational health and safety, and building defects which posed practical problems such as doors having handles and locking properly. These issues rightly took priority to ensure the health and safety of staff and clients. However, they resulted in the collaboration issues simply being put aside by the managers and HPs. The existence of the project and the presence of the research officer did raise awareness of the value of IPP over time as the practical ‘teething problems’ settled.

It was apparent very early that engaging people to come to the research focus groups would be challenging. Initially, this was thought to be because staff were still familiarising themselves to the new space (and ‘organising hooks on doors’ so to speak), and that the discussion of IPP was being presented to them too early. However, upon further reflection, as time proceeded, and with the observations of territorial behaviours, this unwillingness to participate was considered to be in response to feeling insecure and/or unsure about their new work structure. Given the degree of change that these people had experienced, it is reasonable that the HPs were not yet ready to work towards IPP between the co-located agencies. Their priority at that stage was in ensuring more basic needs were met, such as familiarising themselves with the new building and changed structures within the Centre.

## Discussion

Our research found that, although IPP often exists within services, inter-agency integration, teamwork and collaboration within an IPP context is a more complex and elusive task. Co-location of multiple health services must be planned and resourced carefully in order for IPP goals to be achieved. Although there is evidence of successful approaches to collaboration among agencies with independent governance [18,19], what was evident in this setting was that governance arrangements could have received greater focus as part of the move to the Centre. This may have minimised future cultural and operational problems. Clear memoranda of understanding between the services were likely a developing process as part of the move into the Centre. Others have also found that integration of vision, values and practice is often difficult to achieve [20]. As McDonald et al. [21] explain, “community-based health services that cross organisational boundaries add a layer of complexity to interprofessional relationships…[which] has affected the level of trust and mistrust” (p.63). Our study has highlighted a number of reasons why this mismatch occurs. These reasons include infrastructural arrangements, territorial behaviours, unwillingness to collaborate as an initial response to co-location, and striving to meet basic needs first within new and changed arrangements. An Australian study of teamwork competencies, as defined by managers, rated, “commitment to working collaboratively, commitment to the organization and commitment to a quality outcome” [22] (p.22) as the most important motives impacting on team performance. This was certainly apparent in the rhetoric of our focus groups; yet there appeared to be other structural issues that hindered the realisation of team competencies in practice.

A systematic review by Cameron & Lart [23] examined the factors promoting and obstacles hindering joint working in the NHS/social services interface. It offers some interesting insights and parallels to the GP Plus Centre experience. They state that, although co-location can increase opportunities for communication across different services, “different agendas and basic interests…can make agreement on action at a strategic level difficult to achieve” [23] (p.11). They highlight a range of organisational and governance factors that are important for collaboration, such as: the importance of clear aims that are understood and accepted by all those involved; regular formal and informal communication between services to promote more timely and appropriate referrals between them; clarity of roles and responsibilities, and therefore expectations; strong management to create clear lines of responsibility and accountability; and adequate resources and personnel to undertake collaboration [23] (pp.11-13). The cultural and professional obstacles identified by Cameron and Lart’s review included negative assessments or professional stereotypes held by each agency or professional group; different professional philosophies or ideologies; and contextual issues such as the political climate, constant re-organisation and financial uncertainty. They emphasised that trust and respect, which are key to the success of joint working, need time for workers to develop, and that joint training is a useful strategy for helping workers to understand each other’s roles and responsibilities [23] (pp.13-15). This view is confirmed by others [20]. Ayoko et al. [14] recommended training for service leaders in managing workers’ territorial behaviours, given the impacts on workers’ attitudes, emotions and outcomes for teams [see also [24-27]].

The important positive role played by a shared information management system has also been noted. Horvitz-Lennon et al. [24] reported that the mental health sector, for example, has lagged and that, “most people with mental illnesses are cared for in practices whose information systems are under-developed or poorly integrated with general health practices…[and that] information exchange is also constrained by a complicated array of privacy rules and overzealous attitudes by mental health providers” (p.663). Of concern, Horvitz-Lennon et al. [24] noted the separation between the mental health care sector and the general health care sector as a key contributor to higher burden of physical health problems in the mental health population. One aim of the GP Plus initiative was to overcome such barriers and promote a more integrated and streamlined approach to care for people with mental health concerns [1].

The results of our study show that each of the above necessary components for building collaborative practice were not present, or only partially developed in the GP Plus setting. Services had moved into the building and brought their own cultures, information management systems and ways of working with them. The structure of the building also impeded them from developing effective collaborative working relationships with each other. There was no established forum or shared training program where workers could come together to meet or develop a better understanding of each other’s profession or service. Some of the services (especially the mental health services) were also experiencing and preoccupied with ongoing internal reorganisation. This likely disengaged them further from the goals of the GP Plus and the IPP project.

Salmon [25] looked at the challenges of multi-agency collaboration and emphasised that, “barriers arise from arguments about the theoretical basis for such work, professional perceptions and territories, and the nature of vulnerable client groups” (p.157). Salmon continued: “Also important are the guidelines and definitions that establish the mandate or each agency, their target populations and eligibility requirements, budgets and programmatic reporting cycles” [25] (p.157). This suggests that structural and governance issues are also important to address. These challenges were evident in the GP Plus setting as part of the lack of collaboration in many clients’ care across the services They were also demonstrated by the behaviours observed in the first two themes and their consequences for IPP.

Martinson & Holcomb’s [26] large study, based on site visits to health and welfare services in 17 cities in 13 American states, found a range of benefits and challenges of co-location and integration of services. They found that these processes, “created a more complex system for managers, front-line staff, and clients to navigate” [26] (p.11). Other issues identified were the challenges of establishing working relationships with agencies where there had been no prior interaction. This was part of a larger challenge of the time needed to work out which agency ultimately assumed responsibility for clients within the new complex service system [see also 3]. Martinson & Holcomb [26] also stated that, “the range of issues including lack of leadership, lack of perceived benefits, conflicting goals, and different accountability systems” (p.17) led to only limited success in efforts to coordinate services. These structural issues were also evident in our study, demonstrated by barriers to clear governance and information sharing, and the nature of the building’s physical infrastructure.

Williams, Shore & Foy [27] studied co-location of mental health and primary health care services across three models in the United States. They found that co-location on the same floor with common hallways and patient reception increased communication between workers, giving immediate and convenient access across teams and increased comfort, mutual respect, and trust. In our study, HPs had only limited opportunities to run into other staff, given that the elevator could be perceived to have acted as a time tunnel, or Tardis (from the television series Dr Who), in which such contact might be minimal. The extensive research conducted by Moos [28] demonstrates the importance of considering the physical aspects of the setting and their impact on the culture and practice that occurs within it.

Sloper [29] argued that health professionals are rendered ill-prepared for IPP. Their review of the literature found that interprofessional programs of continuing education can help to remove barriers to collaboration and that co-location in shared offices or the same building, “increases opportunities for communication between staff, promoting understanding and information sharing” [29](p.576). We did not find this, likely because of a number of structural issues that were not realised in the planning of the building we researched. This highlights that there are many dynamic processes that need to be considered for co-location to truly work in practice. Co-location is more than just looking at where we place individuals in the physical sense; there is a need to manage governance, cultural and professional boundaries which can block the channels of communication and negotiation required for collaborative practice [30].

We found similarly that coordination and collaboration do not happen on their own, that co-location is not just about the bricks and mortar. It is also about strategies to bring people together in a meaningful way. Sloper [29] found that, “a diverse range of professional groups working together was also associated with higher levels of innovation in patient care” (p.575). However, Ginsburg [3] stressed that, “the reported benefits often result from more than just sharing of space, since they reflect specific strategies that co-located practices use to improve coordination of care” (p.4). Co-location does not necessarily mean people will talk to each other or improve their working relationships. In the absence of dedicated strategies, we found that the reverse can occur, and services can become more territorial and isolated from each other. Ginsburg [3] emphasised that significant interprofessional issues emerge from co-located services’ differences in philosophies, cultures and practice styles and approaches and that when, “such differences are not addressed directly, they could create major barriers to effective care and communication” (p.7). Taylor-Seehafer [30] explained that, “when health professionals collaborate, the focus becomes the client (or practice) rather than the individual providers (or institution)” (p.387). However, for this to occur, according to our observations, the client usually had to have complex needs. This demonstrates the need to work together for the benefit of the patient, regardless of their level of complexity, and to consider structural, managerial, professional and cultural issues.

While mention has been made here of the lack of a united governance structure for the newly developing Centre, as potentially hindering the integration process, Chreim et al. [19] made a case to the contrary in reporting greater sustainability and resilience of agency integration by building a “winning coalition of agents” (p187), rather than a stand-alone leader. They also stressed the need for dedicated resources in the form of a coordinator or research officer as, “a crucial enabler of the change” process (p198). Unfortunately, in the case reported in this paper, the Manager of the GP Plus site made a strong contribution to the coordination of and support for the project. However,, in retrospect, it was unrealistic to expect the Manager responsible at the time for establishing the new Centre, to totally fulfil this role, particularly in the early stages of setting up services in a new building. Again timing was part of the problem here along with insufficient dedicated resources for enhancing integration. Likewise, Hendy and Barlow [31] cautioned against vesting responsibility for change efforts in a few individuals. Change is everyone’s business.

This study has a number of limitations. The length of the observations was somewhat short (10 months) and was not performed systematically, relying on the reflective notes of the research officer. General practitioners, an important member of a coordinated team approach to care, were also not included because they were not located in the Centre. Follow-up observations and focus groups were not undertaken at a later time period, once services were more settled into the building. However, feedback to participants formed part of the action research process during the focus group cycles.

## Conclusions

Our research observations confirm many of the issues that are already known as important challenges for successful co-location of services and actualisation of IPP. However, our research provides further evidence for and description of the physical facility and the accompanying infrastructure and behavioural issues that can impact on the success of realising integration and IPP values and practice in a centre comprising multiple agencies. For co-location to incorporate effective IPP, IPP needs to be a central focus of planning rather than an afterthought or part of the model to be developed once all the doors are hung and the furniture arranged. In the GP Plus setting, IPP got lost in the planning somewhere along the way; they were overtaken by operational issues that kept coming up. The experience of this setting demonstrates that dedicated staff and resources are needed in order to keep carriage of IPP, as new models of collaborative and co-located services are developed. This experience also shows that establishing IPP within newly co-located services is perhaps a process that needs time to develop, as part of teams building trust with each other in new circumstances, in order to eventually build a new culture for the Centre. It shows that health service systems are complex, with competing priorities, increasing service demands, and differing strategic plans and performance indicators to meet. This coupled with the tendency for policy makers to move on to the next issue of focus, and to shift resources in the process, means that adequate time and resources for IPP are often overlooked. Perhaps one way forward is for universities where future health professionals train and health services where they go out to on placement and eventually become part of the workforce, to work together more. Dedicated resources to have shared education or placement of students may be one initiative worthy of greater consideration in the planning of such centres and in the planning of university courses.

## Competing interests

The authors declare that they have no competing interests.

## Authors’ contributions

SL drafted the manuscript. AL collected the data. SL and AL analysed the data. All authors participated in the design of the study and the reference group that informed the analysis and interpretation of the data. All authors read and approved the final manuscript.
